# Diagnostic utility of patient history, clinical examination and screening tool data to identify neuropathic pain in low back related leg pain: a systematic review and narrative synthesis

**DOI:** 10.1186/s12891-020-03436-6

**Published:** 2020-08-10

**Authors:** Jai Mistry, Nicola R. Heneghan, Tim Noblet, Deborah Falla, Alison Rushton

**Affiliations:** 1 St Georges Hospital NHS Foundation Trust, London, UK; 2 Centre of Precision Rehabilitation for Spinal Pain, School of Sport, Exercise and Rehabilitation Sciences, College of Life and Environmental Sciences, University of Birmingham, Birmingham, UK

**Keywords:** Neuropathic pain, Low back related leg pain, Diagnosis, Systematic review

## Abstract

**Background:**

Low back-related leg pain (LBLP) is a challenge for healthcare providers to manage. Neuropathic pain (NP) is highly prevalent in presentations of LBLP and an accurate diagnosis of NP in LBLP is essential to ensure appropriate intervention. In the absence of a gold standard, the objective of this systematic review was to evaluate the diagnostic utility of patient history, clinical examination and screening tool data for identifying NP in LBLP.

**Methods:**

This systematic review is reported in line with PRISMA and followed a pre-defined and published protocol. CINAHL, EMBASE, MEDLINE, Web of Science, Cochrane Library, AMED, Pedro and PubMed databases, key journals and the grey literature were searched from inception to 31 July 2019. Eligible studies included any study design reporting primary diagnostic data on the diagnostic utility of patient history, clinical examination or screening tool data to identify NP in LBLP, in an adult population. Two independent reviewers searched information sources, assessed risk of bias (QUADAS-2) and used GRADE to assess overall quality of evidence.

**Results:**

From 762 studies, 11 studies were included. Nine studies out of the 11 were at risk of bias. Moderate level evidence supports a cluster of eight signs (age, duration of disease, paroxysmal pain, pain worse in leg than back, typical dermatomal distribution, worse on coughing/sneezing/straining, finger to floor distance and paresis) for diagnosing lumbosacral nerve root compression, demonstrating moderate/high sensitivity (72%) and specificity (80%) values. Moderate level evidence supports the use of the StEP tool for diagnosing lumbar radicular pain, demonstrating high sensitivity (92%) and specificity (97%) values.

**Conclusions:**

Overall low-moderate level evidence supports the diagnostic utility of patient history, clinical examination and screening tool data to identify NP in LBLP. The weak evidence base is largely due to methodological flaws and indirectness regarding applicability of the included studies. The most promising diagnostic tools include a cluster of 8 patient history/clinical examination signs and the StEP tool. Low risk of bias and high level of evidence diagnostic utility studies are needed, in order for stronger recommendations to be made.

## Background

One of the most prominent causes for worldwide disability is chronic pain, and up to a fifth of those with chronic pain have neuropathic pain (NP) [1]. NP is defined by the International Association for the Study of Pain (IASP) as “pain arising from a disease or lesion of the somatosensory nervous system” [2]. It has been estimated that up to 1 in 10 people with chronic pain have NP, this is according to point prevalence estimates obtained from different time points between 2004 and 2012 [3]. NP is particularly common in those with low back related leg pain (LBLP) [4], with point prevalence estimates, taken between 2009 and 2012, and ranging between 19 and 80% [5]. The annual direct medical costs associated with NP in LBLP is estimated to be approximately £270 million in the UK alone [6], with the current figure likely to be higher.

LBLP is considered primarily neuropathic in nature when neural tissue in the low back is compromised (e.g. nerve root, dorsal root ganglion), commonly referred to as sciatic or lumbar radicular pain [5]. However, LBLP is not always neuropathic in nature. LBLP can manifest as a result of the involvement of non-neural structures (e.g. muscle, ligament, disc) in the lumbar spine (which similarly can refer pain into the leg); termed as referred pain and commonly associated with nociceptive pain [5]. However, it is well understood that pain does not simply present dichotomously but as a complex interaction of numerous pain mechanisms, as depicted in research investigating the neurobiological basis of lumbar radiculopathy, where NP, ischaemic and mechanical pain mechanisms were found to coexist [7].

The importance of identifying the presence of NP in LBLP is related to ensuring appropriate treatment intervention. The National Institute for Health and Care Excellence (NICE) guidelines for LBP with sciatica [8] recommend that the pharmaceutical management of sciatica is to conform with the NICE guidelines for NP [9]. Pain medication targeted at treating the underlying pain mechanisms is advocated as more effective than those that target a disease entity [10].

There is no gold standard to diagnosing NP in LBLP, furthermore there is no gold standard for diagnosing NP [11]. Screening tools to identify NP in LBP have been developed and validated, such as the Standardised Evaluation of Pain questionnaire (StEP) [12], PainDetect [13] and the Leeds Assessment of Neuropathic Symptoms and Signs (LANSS) [14]. However, these tools are yet to be validated in identifying NP in LBLP and, the literature regarding superiority of one over the other is varied and conflicting [13, 15]. Similarly, research investigating the use of patient history and clinical examination items to diagnose NP in LBLP is lacking and inconclusive [16, 17]. Two separate studies have devised a list of clinical indicators using patient history and clinical examination items to identify peripheral NP in patients with or without leg pain [18] and in lumbosacral nerve root compression [19]. The derived lists share one common item - *pain distributed in a dermatomal pattern*. However, these studies must be observed with caution as items were considered in a cluster and the phenomena of interest in both studies are differently defined and thus difficult to compare directly. To date there has been no systematic review investigating the diagnostic utility of clinical indicators (patient history, clinical examination and screening tools) to identify NP in LBLP.

### Objective

To evaluate the diagnostic utility of patient history, clinical examination and screening tool data in order to identify NP in adults presenting with LBLP.

## Methods

### Design

A systematic review was completed in accordance with a published study protocol [20]. The protocol was informed by the The Cochrane Handbook for Diagnostic Test Accuracy studies and the Centre for Reviews and Dissemination [21] and the Preferred Reporting Items for Systematic Reviews and Meta-Analysis-Protocols (PRISMA-P) checklist [22]. The systematic review is registered with PROSPERO (CRD42019140861). No changes were made to the original protocol [20].

### Eligibility criteria

The Sample, Phenomenon of Interest, Design, Evaluation, Research Type (SPIDER) guidelines were adopted to format and structure the eligibility criteria [23].

#### Inclusion criteria

(S) Sample: adult participants with LBLP.

(PI) Phenomenon of Interest: clinical indicators that identify NP in LBLP.

(D) Design: non-experimental cross-sectional study designs are the ideal design for investigating diagnostic accuracy [24], and therefore optimal for this study. However, other study designs were eligible for inclusion if the study presented primary diagnostic accuracy data.

(E) Evaluation: studies investigating the validity of clinical indicators to identify NP in LBLP.

These clinical indicators included:

- Patient History items (e.g. aggravating factors, pain location, pain description)
- Clinical examination items (e.g. neurodynamic testing, neurological examination, range of movement)
- Screening tools (e.g. LANSS, StEP)

(R) Research type: quantitative or mixed methods (requires relevant quantitative findings of results)

#### Exclusion criteria

- Not written in English
- Studies that did not compare an index test (patient history and/or clinical examination and/or screening tools) against a reference standard to identify NP in LBLP [20]

### Information sources

Two independent authors (JM, TN) independently searched pre-identified electronic databases (searched from inception to 31 July 2019), key journals and grey literature.

Searches comprised of:

- Electronic databases: CINAHL, EMBASE, MEDLINE, Web of Science, Cochrane Library, AMED, Pedro and PubMed
- Key journals: Musculoskeletal Science and Practice, PAIN, European Journal of Pain, The Journal of Pain and The Clinical Journal of Pain
- Grey literature: British National bibliography, OpenGrey and EThOS

### Search strategy

The search was highly sensitive, devised in collaboration with all authors and a specialist librarian [20]. The key terms used for the search were: Diagnostic validity, Patient history, Clinical examination, Screening tool, Neuropathic pain and LBLP.

For the above search terms a list of synonyms and truncations were generated to maximise search inclusion. Key terms were formatted as per the requirements of each specific database in order to retrieve the maximum number of relevant articles. See example of search terms inputted into database (Box 1).

### Study records

#### Data management

Endnote Version X8 (Clarivate Analytics) software programme was used for data management [20]. Abstracts and full texts were compiled and duplicates were removed.

#### Selection process

Two reviewers (JM, TN) conducted a two staged selection process, independently. Firstly, screening of titles and abstracts was completed using the eligibility criteria. Secondly, full texts of prospective studies were obtained and then assessed for eligibility. Any disagreements between reviewers throughout the selection process were discussed and if a solution was not achieved then a third reviewer was consulted (AR). Agreement throughout the selection process between reviewers was measured using the kappa statistic [25].

#### Data collection and data items

The data extraction document was piloted and subsequently used without any modifications required, independently, by the two reviewers (JM, TN). The third reviewer (AR) was again used to settle any disagreements as well as to ensure quality by independently reviewing data extracted.

Extracted data items consisted of: title, author, publication date, study design, participant age, participant gender, participant comorbidities, index test, comparator test, reference standard, sensitivity, specificity, likelihood ratios (LRs) and positive predictive values (PPVs).

### Risk of bias

The Quality Assessment of Diagnostic Accuracy Studies 2 (QUADAS-2) tool was used as it is a recognised tool for assessing risk of bias (RoB) in diagnostic accuracy studies [26]. The four domains of the QUADAS-2 tool (patient selection, index test, reference standard and flow and timing) were independently assessed and judged by each reviewer (JM, TN) as ‘high’, ‘low’ or ‘unclear risk’. Reviewers then provided an overall verdict regarding bias of the studies assessed, ‘at RoB’ or ‘low risk’, if a study was judged as “high risk” or “unclear risk” in one or more domains then an overall judgement of “at RoB” was made [26]. The third reviewer (AR) was used to settle disagreements if consensus was not achieved between the two reviewers (JM, TN) on discussion. Furthermore, agreement was assessed between the reviewers (JM, TN) using Cohen’s k.

### Summary measures

Summary measure tables were developed using the primary diagnostic data (sensitivity, specificity, LRs and PVs) retrieved from the included studies. Where data were not available the lead author (JM) used the raw data to calculate the missing results, using the formulae recommended by Akobeng [27]. Sensitivity and specificity cut of points were graded as low (≤50%), low/moderate (51–64%), moderate (65–74%), moderate/high (75–84%) and high (≥85%) as highlighted in the study protocol [20].

### Data synthesis

Heterogeneity was explored in relation to study design, population, comparable diagnostic data and reference standard to dictate the possibility of doing a meta-analysis. The data extraction form was used to compare study design, population and reference standards between studies and the summary measure tables were used to explore comparable diagnostic data. As stated in the study protocol [20], in the event that a meta-analysis was not possible a narrative synthesis would be conducted [28].

### Confidence in cumulative evidence

The Grading of Recommendations, Assessment, Development and Evaluations (GRADE) was used to assess the level of evidence; the GRADE method was adapted for diagnostic accuracy research (Table 1) [29]. The reviewers (JM, TN) assessed each included study according to five downgrading factors (RoB, inconsistency of evidence, indirectness of evidence, imprecision of results and publication bias) in order to assign GRADE ranking. The GRADE ranking process started with assigning an initial level of quality of evidence based on study design (cross sectional and cohort design considered high quality, any other design considered low quality) and then assessing the study against the downgrading factors to assign a final judgement on level of evidence [20]. Publication bias was suspected in situations where evidence was derived from a number of small studies.

**Table 1:** Modified GRADE for diagnostic accuracy studies

Factors that determine and can decrease the quality of evidence	Explanations and how the factor may differ from the quality of evidence for other interventions
Study design	Cross-sectional or cohort studies in patients with diagnostic uncertainty and direct comparison of test results with an appropriate reference standard (best possible alternative test strategy) are considered high quality and can move to moderate, low or very low depending on other factors.
Risk of bias (limitations in study design and execution)	Representativeness of the population that was intended to be sampled. Patient selection: consecutive or random sample of patients enrolled? Case-control design avoided? Did the study avoid inappropriate exclusions? Independent comparison with the reference standard. All enrolled patients should receive the index test and the reference standard test. Diagnostic uncertainty should be given. Is the reference standard likely to correctly classify the target condition? Flow and timing: was there an appropriate interval between index test(s) and reference standard?
Indirectness Patient population, diagnostic test, comparison test and indirect comparisons of tests	The quality of evidence can be lowered if there are important differences between the populations studied and those for whom the recommendation is intended (in prior testing, the spectrum of disease or co-morbidity); if there are important differences in the tests studied and the diagnostic expertise of those applying them in the studies compared to the settings for which the recommendations are intended; or if the tests being compared are each compared to a reference (gold) standard in different studies and not directly compared in the same studies. Panels assessing diagnostic tests often face an absence of direct evidence about impact on patient-important outcomes. They must make deductions from diagnostic test studies about the balance between the presumed influences on patient-important outcomes of any differences in true and false positives and true and false negatives in relationship to test complications and costs. Therefore, accuracy studies typically provide low quality evidence for making recommendations due to indirectness of the outcomes, similar to surrogate outcomes for treatments.
Important Inconsistency in study results	For accuracy studies unexplained inconsistency in sensitivity, specificity or likelihood ratios (rather than relative risks or mean differences) can lower the quality of evidence.
Imprecise evidence	For accuracy studies wide confidence intervals for estimates of test accuracy, or true and false positive and negative rates can lower the quality of evidence.
High probability of Publication bias	A high risk of publication bias (e.g., evidence only from small studies supporting a new test, or asymmetry in a funnel plot) can lower the quality of evidence.

## Results

### Study identification

**Fig. 1:**
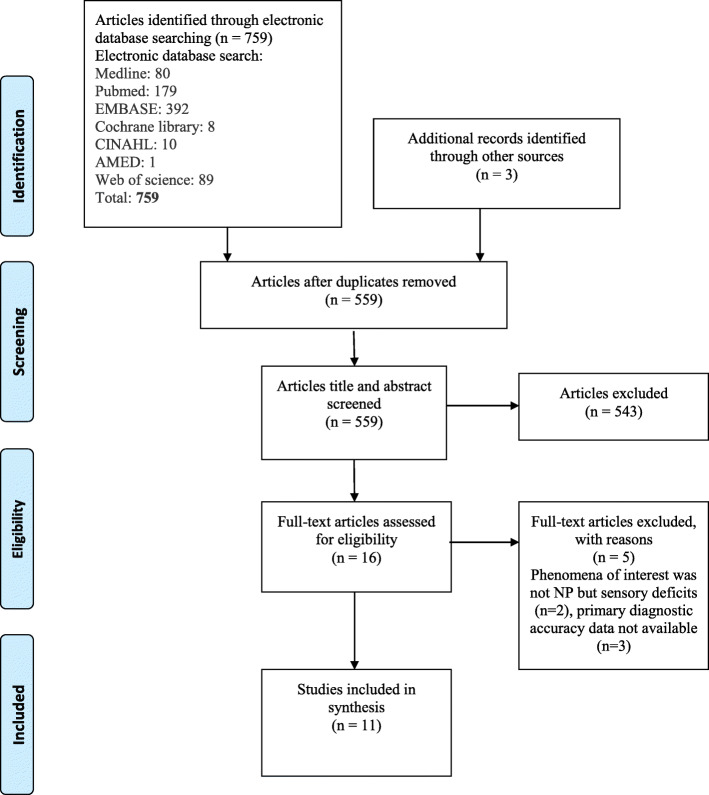
Study selection flow diagram

Initial searches using electronic databases and additional sources resulted in 762 studies being retrieved. Following duplicate removal and title and abstract screening, 16 studies remained for full text review. On completion of full text screening, 5 studies were excluded and a subsequent 11 studies were included for analysis (Fig. 1).

### Study characteristics

Table 2 depicts the characteristics of the 11 included studies.

**Table 2:** Characteristics of included studies table

Author	Study design	Phenomena of interest	Inclusion & exclusion criteria	Population	Index test	Reference standard
Capra et al., 2011 [16] Italy	Cross-sectional observational study	Sciatica with or without lumbar pain	Inclusion: 16–85 years, acute or recurrent sciatica (located distal to the knee), undergone MRI. Exclusion: diabetes, neoplasia, spinal cord disease, workers compensation, prior surgery, peripheral neuropathy, retroperitoneal pathology.	*n = 2352 (Female: *n* = 1120, Male: *n* = 1232) Mean (*SD) age: 49.22 (14.68)	Straight leg raise	*MRI
Gudala et al., 2017 [30] India	Cross-sectional observational study	*CLBP with or without leg pain	Inclusion: > 18, CLBP > 3 months, understand Hindi. Exclusion: diabetes, cancer, chronic pain conditions, pregnant, incomplete data.	*n* = 215 (Female: *n* = 104, Male: *n* = 111) Mean (SD) age: 46.6 (13.9)	*S-DN4, ID Pain, painDETECT questionnaire, *S-LANSS	Physician opinion
Lin et al., 2017 [17] Taiwan	Cross-sectional observational study	Lumbar lateral stenosis involving L5 nerve root	Inclusion: back pain with or without leg pain lasting > 3 months, corresponding lesion on MRI (central spinal stenosis, lateral recess stenosis, foraminal stenosis, segmental instability. Exclusion: spinal tumour/infection, Cauda equina syndrome, refused index test, Visual analogue scale < 2, bilateral L5 symptoms. L4/5 foraminal stenosis or L5/S1 central stenosis and those who have pathology not involving the L4/5 or L5/S1 level.	n = 60 (Female: *n* = 38, Male: *n* = 22) Mean (SD) age: 61.37 (Nil reported)	*SQST	MRI: grade 3 lateral stenosis
Poiraudeau et al., 2001 [31] France	Cross-sectional observational study	Sciatica associated with disc herniation	Inclusion: Patients hospitalised for acute or chronic sciatica of mechanical origin. Sciatica defined as: “lumbosacral and lower limb pain, associated or not with paraesthesias and with one of the following conditions: radicular pain below the knee after an L5 or S1 nerve root dermatome; and radicular pain above the knee associated with neurological impairment reflex abolition, muscular weakness or sensory defects in the corresponding radicular area).” (Poiraudeau et al., 2001). Exclusion criteria: LBP without sciatica, radicular pain in a dermatome other than L5 and or S1, systemic lumboradicular pain tumour, infectious or inflammatory disease, prior lumbar surgery, uncontrolled psychiatric disorder.	*n* = 78 (Female: *n* = 45, Male: *n* = 33) Mean (SD) age: 50 (16)	Bell’s test, *HE test, Lasegue signs, Crossed Lasegue sign	MRI, CT, saccoradiculography
Scholz et al., 2009 [12] USA	Cross-sectional observational study	NP in LBP (radicular)	Inclusion: CLBP, pain duration ≥3 months, Visual analogue scale > 6, age ≥ 18 . Exclusion criteria: severe medical or psychiatric illness, painful disorder or neurological disease that might have interfered with the pain assessment, local infection	*n* = 138 (Female: *n* = 78, Male: *n* = 60) Mean (SD) age: 45 (Nil reported)	* StEP tool	Independent physician clinical diagnosis
Smart et al., 2012 [18] Ireland	Cross-sectional observational study	Peripheral NP in patients with or without leg pain	Inclusion: > 18, LBP with or without leg pain, those with a dominance of peripheral NP (deemed through a Delphi consensus list). Exclusion: Patients with a history of diabetes or central nervous system injury, pregnancy or non-musculoskeletal LBP	n: 102 (Female: *n* = 53, Male: *n* = 49) Mean (SD) age: 44 (13.1)	Cluster of subjective/ objective indicators	Clinical judgement
Trainor et al., 2011 [32] England	Cross-sectional observational study Pilot study	Upper/mid lumbar nerve root compression	Inclusion: lumbosacral radicular pain, defined as: pain radiating unilateral/bilateral distal to gluteal crease, pain distribution in dermatome area/1 or 2 levels above/below Exclusion: cervical/thoracic pain, Red flags, spinal pathology or systemic illness, recent quads injury or unable to lie in test position.	n: 16 (Female: n = 7, Male: *n* = 9) Mean (SD) age: 49 (Nil reported)	Slump knee bend	MRI
Urban et al., 2015 [33] Canada	Cross-sectional observational study	NP in lower limb	Inclusion: LBP with or without leg pain, 25 years or >, English speaking, suitable for complete neuro exam, conservatively managed. Exclusion: Previous back surgery, systemic illness or central condition.	n: 21 (Female: n = n/a, Male: n = n/a) Mean (SD) age: Nil reported	Slump	Standard clinical Assessment
Verwoerd et al., 2014 [34] Netherlands	Cross-sectional observational study	Lumbosacral nerve root compression	Inclusion: 18–65 age, diagnosed, by neurologist, “incapacitating lumbosacral radicular syndrome, 6–12 weeks. Exclusion: Cauda equina, unable to resist against gravity muscle strength, previous spinae surgery, sever comorbidity, pregnancy, similar episode in last 12 months.	n: 395 (Female: *n* = 147, Male: *n* = 248) Mean (SD) age: 42.8 (10)	History taking	MRI
Vroomen et al., 2002 [19] Netherlands	Cross-sectional observational study	Lumbosacral nerve root compression	Inclusion: pain that warrants bed rest for 14 days, new onset LBLP (distal to gluteal crease). Exclusion: spinal surgery, pregnancy, severe comorbidity, contraindication to MRI.	n: 274 (Female: *n* = 139, Male: *n* = 135) Mean (SD) age: 46 (Nil reported)	History and Physical examination	MRI
Walsh et al., 2009 [14] Ireland	Cross-sectional observational study	LBLP	Inclusion: unilateral LBLP, 18–70 age, speaks English. Exclusion: absence of unilateral LBLP, serious pathology, spinal surgery or neurological disease, unbale to tolerate testing positions.	n: 45 (Female: n = 23, Male n = 22) Mean (SD) age: 46 (11)	Nerve palpation	SLR + slump

*n number, *SD standard deviation, *MRI Magnetic resonance imaging, *CLBP Chronic low back pain, *S-DN4 Self-completed douleur neuropathique 4, *S-LANSS Self-completed Leeds Assessment of neuropathic Symptoms and Signs, *SQST standardised qualitative sensory testing, *HE Hyperextension, *StEP Standardized Evaluation of Pain

#### Study design

All studies used cross sectional observational study designs. One study was a pilot study with a cross sectional observational design [32].

#### Participants

In total 3908 participants were investigated across the 11 included studies, with ages ranging 30–70 years. One study did not report the age of participants [33]. The phenomena of interest varied significantly between studies; two studies investigated lumbosacral nerve root compression [19, 34], one study investigated participants with upper/mid lumbar nerve root compression [32] and another looked specifically at L5 lateral stenosis [17]. Two studies investigated peripheral NP and chronic low back pain respectively [18, 30] with and without leg pain, whereas Capra et al. [16] investigated sciatica with or without lumbar pain. Poiraudeau et al. [31] investigated participants with sciatica associated with disc herniation and Walsh et al. [35] studied those with LBLP. Finally, Urban et al. [33] investigated participants with NP in the lower limb and Scholz et al. [12] investigated participants with radicular pain.

#### Index test

Two studies investigated the diagnostic validity of NP screening tools (S-DN4, ID Pain, painDETECT questionnaire, S-LANSS and StEP tool) [12, 30]. One study investigated the diagnostic accuracy of patient history data [34], whilst two studies investigated both patient history data and clinical examination data [18, 19]. Finally the remaining six studies investigated the use of clinical examination tests; Straight leg raise (SLR) [16], Slump test [33], slump knee bend [32], nerve palpation [35], standardised qualitative sensory testing (SQST) [17], and bell test/hyperextension test [31].

#### Reference standard

The most commonly used reference standard test was magnetic resonance imaging (MRI); this was used in six of the included studies [16, 17, 19, 31, 32, 34], one of which used MRI and/or another imaging technique (computed tomography & saccoradiculography) as a reference standard [31]. Four studies used clinical judgement as a reference standard through a clinical examination [12, 18, 30, 33]. Clinical judgement was defined in each of the four studies as; a single physician examination [30], an experienced Rheumatologist, Neurosurgeon and Physiotherapist examination [12], a Consultant in pain medicine and expert Physiotherapist examination [18] and two Orthopaedic manual therapists examination [33]. Years of experience was not specified in any of the four studies. Finally, one study used clinical examination tests as a reference standard; Walsh et al. [35] used the SLR and the slump test as a reference standard.

### Risk of bias

**Fig. 2:**
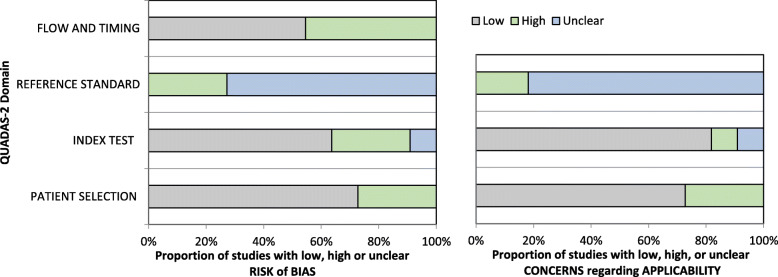
QUADAS 2 RoB assessment findings

Complete agreement was achieved between the two reviewers for assessment of RoB, and thus the third reviewer was not required. Two studies were assessed as low RoB [12, 19], the remaining nine studies were considered at RoB (Table 3, Fig. 2). The primary concerns in relation to the at RoB studies were blinding of index and reference tests/insufficient description of procedures involved in index and reference test (six studies), flow and timing between tests (four studies) and patient selection (three studies). For all of the eleven included studies the reference standard for RoB and applicability was scored largely as unclear. This is because in the absence of a gold standard or clear recommendations/guidelines to diagnose NP in LBLP it is unclear whether the reference standards used in the studies correctly classify the target condition. Table 3 and Fig. 2 depict RoB and applicability concerns for each of the 11 included studies.

**Table 3:**
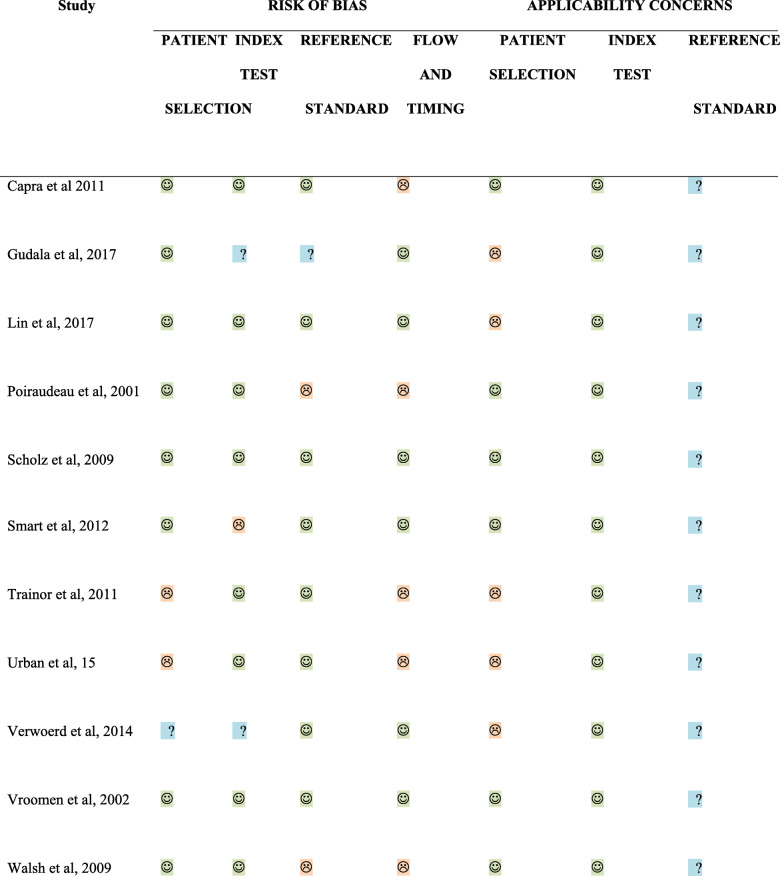
QUADAS 2 RoB assessment findings

### Synthesis of results

A meta-analysis was not completed since there were inconsistencies in the reference standard used between studies. Even amongst the studies that used the same reference standard, differences were highlighted in how it was measured [16, 17]. Furthermore, the majority of studies were considered at RoB making any further statistical analysis equally at RoB. Finally, the number of studies retrieved for screening tools (*n* = 2) and patient history taking (*n* = 3) were limited and the studies investigating a clinical examination test used a wide variety of different tests resulting in insufficient data for pooling. A narrative synthesis was therefore conducted.

### Patient history data

One study, at RoB, investigated patient history data [34] in relation to diagnosing nerve root compression or herniated disc in patients with LBLP. This study investigated 20 separate patient history items (Table 4). Of the 20 items, moderate/high and high sensitivity values in both herniated disc and nerve compression groups were observed for health-related absenteeism (81 and 80% respectively) and in subjective sensory loss (89 and 90% respectively). Having had pain in the same leg previously demonstrated the highest specificity, in both herniated disc and nerve compression groups (90 and 91% respectively). Indirectness of evidence was highlighted as a highly selective population of patients were recruited (Table 5). Using GRADE, there is low quality of evidence to support the use of Verwoerd et al’s [34] patient history indicators in diagnosing nerve root compression or herniated discs (Table 5).

**Table 4:**
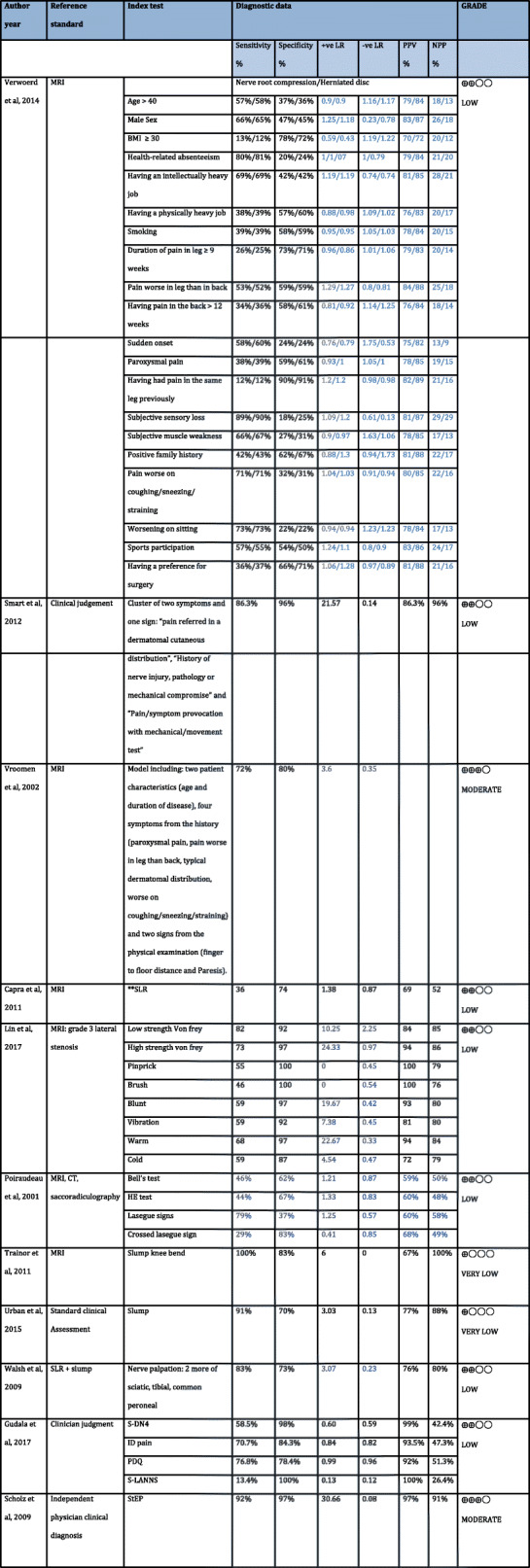
Summary measures table of Patient History data, clinical examination data and screening tool data

*SLR straight leg raise

Figures in blue were calculated by the reviewers of this paper, raw data was used from the original studies

**Table 5:** Grade quality assessment for patient history, clinical examination and screening tool data

				GRADE Quality assessment						
Index test/clinical indicators	Sample size	Studies per index test/clinical indicator	Phenomena of interest	Study design	RoB	Indirectness	Inconsistency	Imprecision	Publication bias	Quality
Verwoerd et al’s, (2014) [13] 20 subjective clinical indicators (see Table 4)	395	1	Lumbosacral nerve root compression	Cross sectional observational – no limitations in study design	Serious RoB (see Table 3 and Fig. 2)	Serious indirectness	No serious inconsistency	No serious imprecision	Undetected	⨁⨁◯◯ LOW ^a,b^
Cluster of two symptoms and one sign: “pain referred in a dermatomal cutaneous distribution”, “History of nerve injury, pathology or mechanical compromise” and “Pain/symptom provocation with mechanical/movement test”	464	1	Peripheral NP in patients with or without leg pain	Cross sectional observational – no limitations in study design	Serious RoB (see Table 3 and Fig. 2)	Serious indirectness	No serious inconsistency	No serious imprecision	Undetected	⨁⨁◯◯ LOW ^a,c^
Model including: two patient characteristics (age and duration of disease), four symptoms from the history (paroxysmal pain, pain worse in leg than back, typical dermatomal distribution, worse on coughing/sneezing/straining) and two signs from the physical examination (finger to floor distance and Paresis).	274	1	Lumbosacral nerve root compression	Cross sectional observational – no limitations in study design	No serious RoB (see Table 3 and Fig. 2)	Serious indirectness	No serious inconsistency	No serious imprecision	Undetected	⨁⨁⨁◯ MODERATE 3
SLR	2352	1	Sciatica	Cross sectional observational – no limitations in study design	Serious RoB (see Table 3 and Fig. 2)	Serious indirectness	No serious inconsistency	No serious imprecision	Undetected	⨁⨁◯◯ LOW ^a,c^
SQST	60	1	Lumbar lateral stenosis involving L5 nerve root	Cross sectional observational – no limitations in study design	Serious RoB (see Table 3 and Fig. 2)	Serious indirectness	No serious inconsistency	No serious imprecision	Undetected	⨁⨁◯◯ LOW ^a,c,d^
Bell’s test, HE test, Lasegue signs, Crossed Lasegue signs	78	1	CLBP with or without leg pain	Cross sectional observational – no limitations in study design	Serious RoB (see Table 3 and Fig. 2)	Serious indirectness	No serious inconsistency	No serious imprecision	Undetected	⨁⨁◯◯ LOW ^a,c,e^
Slump knee bend	16	1	Upper/mid lumbar nerve root compression	Cross sectional observational design – pilot study	Serious RoB (see Table 3 and Fig. 2)	Serious indirectness	No serious inconsistency	Serious imprecision	Undetected	⨁◯◯◯ VERY LOW 1,3,6,7,8
Slump test	21	1	NP in Lower Limb	Cross sectional observational – no limitations in study design	Serious RoB (see Table 3 and Fig. 2)	Serious indirectness	No serious inconsistency	No serious imprecision	Undetected	⨁◯◯◯ VERY LOW 1,3,7
Nerve palpation: 2 or more of sciatic, tibial, common peroneal	45	1	LBLP	Cross sectional observational – no limitations in study design	Serious RoB (see Table 3 and Fig. 2)	Serious indirectness	No serious inconsistency	No serious imprecision	Undetected	⨁⨁◯◯ LOW ^a,i^
S-DN4, ID pain, PDQ, S-LANNS	215	1	CLBP with or without leg pain	Cross sectional observational – no limitations in study design	Serious RoB (see Table 3 and Fig. 2)	Serious indirectness	No serious inconsistency	No serious imprecision	Undetected	⨁⨁◯◯ LOW ^a,j^
StEP tool	138	1	NP in LBP (radicular)	Cross sectional observational – no limitations in study design	No serious RoB (see Table 3 and Fig. 2)	Serious indirectness	No serious inconsistency	No serious imprecision	Undetected	⨁⨁⨁◯ MODERATE 1,3,11

^a^Downgraded due to being at “high risk” of bias

^b^ Downgraded due to indirectness observed in study due to highly selective population

^c^ Downgraded due to indirectness observed in study as imaging/examination/opinion were used as reference standards all of which are not validated to identify NP in LBLP

^d^ Downgraded due to indirectness observed in study as population comprised of exclusively surgical patients and thus not representative of those managed conservatively

^e^ Downgraded due to indirectness observed in study as reference standards were poorly specified. The use of MRI, CT and saccoradiculography are described without any description of how each will be assessed

^f^ Downgraded to low quality due to study design. This study was a pilot study

^g^ Downgraded due to indirectness observed in study as small population size was not representative of target population

^h^ Downgraded due to imprecision observed in study as wide confidence intervals noted for all measures of diagnostic accuracy. In particular positive predictive value (22–96%)

^i^ Downgraded due to indirectness observed in study as SLR and Slump test were used as a reference standard which are not validated tests to identify NP in LBLP

^j^ Downgraded due to indirectness observed in study as population included those with LBP with or without leg pain which is not consistent with the target population for this review. Also, the questionnaires used in this study were translated into Hindi and yet to be validated. Furthermore, the description of reference standard, physician opinion, was inadequately described and thus indirect

^k^ Downgraded due to indirectness observed in study as equipment needed for the StEP tool are not readily available in clinical practise

### Patient history data and clinical examination data

Two studies investigated both patient history taking and clinical examination findings together [18, 19], in relation to diagnosing peripheral NP in LBP with or without leg pain [18] and in suspected lumbosacral nerve root compression [19]. Smart et al. [18] identified a cluster of three signs and symptoms (pain referred in dermatomal or cutaneous distribution, history of nerve injury, pathology or mechanical compromise, pain/symptom provocation with mechanical/movement tests) which demonstrated a high sensitivity (86.3%) and specificity (96%) (Table 4). This study was considered at RoB as clinicians were aware of the reference standard before issuing the index test. Furthermore, indirectness of evidence was highlighted due the use of clinical judgement as a reference standard without specifying what criteria were used to make this judgement. Using GRADE, there is a low level of evidence to support Smart et al’s [18] cluster of signs and symptoms in diagnosing peripheral NP in LBP (Table 5).

Vroomen et al’s [19] study was deemed to be low RoB. Vroomen et al. [19] identified 8 signs (including patient history and clinical examination signs) which were predictive of lumbosacral nerve root compression demonstrating moderate sensitivity (72%) and moderate/high specificity (80%) (Table 4). This study shared one common item with Smart et al’s [18] cluster; pain referred in a dermatomal distribution. In both instances this indicator was used in association with other indicators, raw data were not available to assess this indicator in isolation. Vroomen et al. [19] used MRI as a reference standard, which has been questioned for its diagnostic validity [36], furthermore this study was investigating lumbosacral nerve root compression which does not necessarily infer NP. Using GRADE, there is a moderate level of evidence to support Vroomen et al’s [19] eight signs in diagnosing lumbosacral nerve root compression (Table 5).

Six studies investigated the use of clinical examination tests in isolation. All six studies were considered at RoB [14, 16, 17, 31–33]. Two studies investigated the diagnostic accuracy of the SLR test for identifying sciatica. Capra et al. [16] found a low sensitivity (36%) and moderate specificity (74%) whilst Poiraudeau et al. [31] found the opposite, moderate/high sensitivity (79%) and low specificity (37%) (Table 4). Indirectness was highlighted in both studies partly due to the use of imaging as a reference standard (Table 5). Using GRADE, there is low level evidence to support the use of the SLR test in diagnosing sciatica. Poiraudeau et al. [31] also investigated three other tests (Bell’s test, HE test, Crossed lasegue test) all of which demonstrated low or low/moderate sensitivity and specificity values (Table 4), expect for moderate/high specificity found for the crossed lasegue test (83%). Using GRADE, there is low level evidence to support Bell’s test, HE test and Crossed lasegue test in diagnosing sciatica (Table 5).

The slump knee bend [32] was found to have high and moderate/high sensitivity and specificity values (100, 83%) diagnosing upper/mid lumbar nerve root compression, similarly the slump test [33] had high and moderate/high sensitivity and specificity values (91, 78%) diagnosing NP in LBLP (Table 4). Low sample sizes were characteristic of both these studies, with one being a pilot study [32]. Using GRADE, there is very low evidence to support the diagnostic utility of the slump knee bend and slump test in diagnosing upper/mid lumbar nerve root compression and NP in LBLP respectively (Table 5).

Nerve palpation was found to have moderate/high sensitivity (83%) and moderate specificity (73%) in identifying LBLP [35], the SLR and slump tests were used as reference standards which led to serious indirectness being highlighted in this study. Low quality of evidence supports the use of nerve palpation in diagnosing LBLP, following the use of GRADE (Table 5). SQST was found to have low/moderate sensitivity (62%) and high specificity (95%) when detecting lumbar lateral stenosis of the L5 nerve root [17] (Table 4). However, indirectness of evidence was highlighted as the participants recruited into this study were all surgical patients and therefore not fully representative of the target population for this review. Using GRADE low level of evidence supports the use of SQST in diagnosing lumbar lateral stenosis of the L5 nerve root (Table 5).

### Screening tool data

One study investigated four screening tools; S-DN4 (Self-completed douleur neuropathique, ID Pain, PDQ (painDETECT questionnaire) and S-LANSS (Self-completed Leeds Assessment of Neuropathic symptoms and Signs) [30] to identify NP in LBP. Three of the screening tools were identified as having a range of low/moderate to high sensitivity and specificity values; 58.5% & 98% (S-DN4), 70.7% & 84.3% (ID Pain), 76.8% & 78.4% (PDQ) (Table 4). However, the S-LANSS was identified as having a low specificity of 13% (Table 4). This study was deemed at RoB as patient applicability was compromised, this was partly due to the recruitment of patients with LBP with or without leg pain which is not consistent with the target population for this review. Furthermore, the reference standard, clinical judgement, was not adequately described and thus subject to bias. Additionally, this study was completed in a different language and cross-cultural validation cut of points used are yet to be validated. Using GRADE, there is low level of evidence to support the diagnostic utility of the S-DN4, ID Pain, painDETECT and S-LANSS tools in diagnosing NP in LBP (Table 5). The StEP tool [12] was found to have a high sensitivity (92%) and specificity (97%) when diagnosing lumbar radicular pain, this evidence was found to be of low RoB. Using GRADE, there is moderate level of evidence to support the diagnostic utility of the StEP tool in diagnosing lumbar radicular pain (Table 5).

## Discussion

This is the first systematic review to investigate the diagnostic utility of patient history, clinical examination and screening tool data to identify NP in LBLP. The results of this review highlight low-moderate level evidence supporting the diagnostic utility of patient history, clinical examination and screening tool data to identify NP in LBLP. The most promising diagnostic tools include a cluster of 8 patient history/clinical examination signs and the StEP tool where moderate level evidence was found following the use of GRADE. However, the moderate level of evidence supporting these two clinical indicators are reflective of data from single studies and therefore must be observed with caution. Eleven studies were included in this review and only two were at low RoB, therefore the conclusions that can be made from this systematic review are limited, however the findings have led to important recommendations of further targeted research.

In order to effectively investigate the diagnostic utility of clinical indicators to diagnosis NP in LBLP a common reference standard is needed which is used uniformly within the literature and in clinical practice. Secondly, consensus regarding accurate and consistent use of terminology when referring to NP in LBLP (e.g. sciatica, lumbar radicular, LBLP) is needed so that literature can be collated and compared without confusion. Finally, studies investigating diagnostic utility must be at low RoB and a high level of evidence must support the use of the investigated clinical indicators in diagnosing NP in LBLP for recommendations to made based on their findings. To ensure future studies are at a low RoB it is essential that appropriate blinding of both the reference and index tests are carried out, patient population is fully representative of the target population and flow and timing between tests is described in detail and justified.

### Patient history and clinical examination

Patient history indicators to diagnose lumbosacral nerve root compression have been investigated by Verwoerd et al. [34] (low level of evidence), this study found moderate/high sensitivity in; “health-related absenteeism”, high sensitivity in “subjective sensory loss” and high specificity in “having had pain in the same leg previously.” However, there is no further evidence to support these patient history indicators in diagnosing NP in LBLP. Clusters of patient history and clinical examination indicators have been highlighted by two studies in this review demonstrating high sensitivity and specificity in one study [18] and moderate sensitivity and moderate/high specificity in the other [19]. Low quality evidence supports a cluster of three signs and symptoms in diagnosing peripheral NP (pain referred in dermatomal or cutaneous distribution, history of nerve injury, pathology or mechanical compromise and pain/symptom provocation with mechanical/movement tests) [18]. Moderate level of evidence supports the diagnostic utility of a cluster of eight signs in diagnosing lumbosacral nerve root compression (two patient characteristics - age and duration of disease, four symptoms from the history - paroxysmal pain, pain worse in leg than back, typical dermatomal distribution, worse on coughing/sneezing/straining and two signs from the physical examination - finger to floor distance and Paresis) [19]. These two studies share only one common indicator; pain referred in a dermatomal distribution. However, this indicator was included as part of a cluster of signs/symptoms in both studies and therefore the diagnostic validity of this indicator alone is unclear. The 2016 Neuropathic Pain Special Interest Group (NeuPSIG) grading system highlights that in order for NP to be probable or definite pain/sensory signs must follow a neuroanatomically plausible distribution [37], which would encompass a dermatomal pattern, supporting the use of this clinical indicator. Conversely, research investigating entrapment neuropathies has demonstrated an extraterritorial spread of symptoms following mild sciatic nerve compression [38], disputing the use of this indicator. Due to the lack of clarity of the performance of this indicator in isolation and the uncertainty in the literature, the diagnostic utility of this patient history indicator remains unclear.

The SLR was found to have moderate/high sensitivity and low specificity when diagnosing sciatica [31], however on the contrary Capra et al. [16] found the opposite in their study investigating sciatica (low sensitivity and moderate specificity). Overall low level of evidence supports the use of the SLR in diagnosing sciatica. The slump knee bend [32] and slump test [33] were found to have high sensitivity and moderate/high specificity in diagnosing upper/mid lumbar nerve root compression and peripheral NP in the lower limb respectively. Very low level of evidence associated with both these tests were largely due to the small sample sizes used in each study. Evidence to support the use of neurodynamic testing to identify NP in LBLP is conflicting with an increasing body of evidence highlighting the low diagnostic validity of these tests [38].

SQST [17] demonstrated low/moderate sensitivity and high specificity when diagnosing lumbar lateral stenosis involving the L5 nerve root in a study at RoB. The population of patients used were all surgical and therefore not fully representative of the target population for this review, thus the applicability of these findings is poor. There is evidence to support the use of quantitative sensory testing (QST) in diagnosing small fibre nerve degeneration in entrapment neuropathies [39]. However, SQST differs to QST as it describes tests which are inexpensive and accessible within a clinical setting (e.g. coin for testing temperature). Evidence to support SQST to detect small fibre nerve degeneration is limited [40] and yet to be investigated in participants with LBLP. The sensory profiles of those with NP in LBLP is not known and therefore support for SQST in identifying NP in LBLP is inconclusive.

### Screening tools

A range of low/moderate to high sensitivity and specificity values were found for S-DN4, ID Pain and PDQ in a study investigating CLBP with or without leg pain [30]. This study was found to be at RoB and indirectness was observed due to inconsistencies in cross cultural validation. Scholz et al. [12] found high sensitivity and specificity in their study investigating the use of the StEP tool in identifying lumbar radicular pain, this study was at low RoB. Moderate level of evidence supports the diagnostic utility of the StEP tool in diagnosing lumbar radicular pain. However clinical judgement was used as a reference standard which was not adequately described, furthermore clinical judgement is not a validated means to identify NP in LBLP. There is no further research to support the use of the StEP tool in identifying NP in LBLP, further research is needed to support its use.

### Collective synthesis of patient history data, clinical examination data and screening tool data

Collective synthesis of patient history data, clinical examination data and screening tool data

Primary diagnostic data reported in these studies support the use of certain subjective history items, clinical examination items and screening tools, however due to the overall RoB assessment and low level of evidence supporting the use of clinical indicators these results must be observed with caution. Only two studies were reported as low RoB and demonstrated moderate level of evidence supporting the diagnostic utility of a cluster of eight patient history/clinical examination signs and the StEP tool in diagnosing lumbosacral nerve root compression and lumbar radicular pain respectively. However, due to the indirectness of these studies in relation to the central question of this review the diagnostic utility of these indicators in regards to identifying NP in LBLP remains unclear.

Indirectness highlighted in all of the included studies is largely related to the phenomena of interest being investigated and its consistency with the focus of this review in identifying NP in LBLP. Included studies investigated the diagnostic utility of clinical indicators in relation to identifying; lumbosacral nerve root compression, L5 lateral stenosis, sciatica, LBLP and chronic LBP, all of which may imply NP in LBLP but not explicitly. Without appropriately defining in the study that NP in LBLP will be investigated, the above-mentioned titles remain ambiguous. Furthermore, in studies where the phenomena of interest are termed as such that imaging is needed to confirm them, e.g. lateral stenosis, it could be questioned whether this an appropriate approach to identify NP. It is well established that structural abnormalities found on imaging are not always directly correlated with symptom presentation [36]. In cases where sciatica is the phenomena of interest, without specifying the interest of investigating the presence of NP within this presentation, sciatica could also encompass cases where NP is not present, as highlighted by Mahn et al., [41]. This is also the case for studies that investigate LBLP, as a manifestation of LBLP may be pain induced by activation of the nervi nevorum (connective tissue sheaths of the peripheral nerve) which result in increased mechanosensitivity which is deemed largely nociceptive in nature and can occur without NP [39]. Furthermore, pain into the leg originating from the back may also be as a result of non-nervous tissue in the lumbar spine being implicated (such as muscle, ligament, disc) which can follow a somatic referred pattern into the leg [5].

As a result of the indirectness highlighted regarding applicability concerns as well as the highly heterogenous data, the studies have been largely assessed individually and the limited synthesis made between studies have been suggested with caution. Due to the general low level of evidence, high RoB and indirectness of evidence we believe that further research is needed to address the title of this review.

### Strengths and limitations

A strength of this systematic review is it adhered to a pre-defined protocol which enabled robust identification and synthesis of the available evidence. Through the analysis that was carried out, recommendations for future research have been made. In the absence of a gold standard to diagnosis NP in LBLP there is no standardised commonly used reference standard in its place, this is a key limitation to this review. Therefore, the use of imaging, clinical opinion and clinical tests used within the included studies are questioned as it is unclear which reference standard is superior. This in turn results in the interpretation of the primary diagnostic accuracy data generated from these studies being contentious, as the reference standard is subject to debate. Another limitation to this study was that, due to the highly heterogeneous data obtained from the included studies, a meta-analysis was not possible. Furthermore, due to the general low level of evidence supporting the investigated clinical indicators and high RoB owing to a range of reasons (Table 6), the conclusions made from this systematic review are limited. Finally, the moderate level of evidence supporting the two clinical indicators (a cluster of eight patient history/clinical examination signs and the StEP tool) must be observed with caution. The evidence used to support this level of evidence is assessed from individual studies and therefore despite being deemed ‘moderate level of evidence’ (following the use of GRADE) the generalisability to a wider population is poor.

**Table 6:** Reasons for each risk of bias item

Capra et al., 2011 [16]	**Risk of bias** Flow and timing (*high risk)*: high risk due to the time intervals between the reference standard, index test and any other treatment administered was not stated in the study. **Applicability concerns** Reference standard (*Unclear*): unclear as there is no clear gold standard for diagnosing NP in LBLP
Gudala et al., 2017 [30]	**Risk of bias** Index test *(Unclear):* it was not stated if the index test was administered without prior knowledge of reference standard results. Furthermore, the use of Physicians assessment as a gold standard was not supported with any pre-defined threshold. Reference standard *(Unclear)*: it was not stated in the study whether the reference standard was administered without prior knowledge of the index test results. **Applicability concerns** Reference standard (*Unclear*): unclear as there is no clear gold standard for diagnosing NP in LBLP
Lin et al., 2017 [17]	**Applicability concerns** Patient selection *(high risk):* The patient population selected for this study were exclusively surgical patients and therefore not entirely consistent with the target population for this review. Reference standard (*Unclear*): unclear as there is no clear gold standard for diagnosing NP in LBLP
Poiraudeau et al., 2001 [31]	**Risk of bias** Reference standard *(high risk)*: examiner 1 of 3 was involved with initial patient clerking/examination which may have influenced interpretation of reference standard results. Flow and timing *(high risk*): All tests were done on the same day however the time intervals between tests were not specified. **Applicability concerns** Reference standard (*Unclear*): unclear as there is no clear gold standard for diagnosing NP in LBLP
Scholz et al., 2009 [12]	**Applicability concerns** Reference standard *(Unclear*): unclear as there is no clear gold standard for diagnosing NP in LBLP
Smart et al., 2012 [18]	**Risk of bias** Index test *(high risk)*: index test was conducted with knowledge of the results of the reference standard. **Applicability concerns** Reference standard (*Unclear*): unclear as there is no clear gold standard for diagnosing NP in LBLP
Trainor et al., 2011 [32]	**Risk of bias** Patient selection (*high risk*): convenience sampling was used to recruit patients. Flow and timing *(high risk)*: ‘small’ intervals were taken between each examiner conducting the index test (slump knee bend test), which have influenced test result. **Applicability concerns** Patient selection: due to small sample size in this study the applicability to the wider target population is poor. Reference standard (*Unclear*): unclear as there is no clear gold standard for diagnosing NP in LBLP
Urban et al., 2015 [33]	**Risk of bias** Patient selection (*high risk*): convenience sampling was used to recruit patients. Flow and timing *(high risk)*: the index test was completed immediately after the clinical examination (reference standard) which may have influenced the results of the index test. **Applicability concerns** Patient selection: due to small sample size in this study the applicability to the wider target population is poor. Reference standard (*Unclear*): unclear as there is no clear gold standard for diagnosing NP in LBLP
Verwoerd et al., 2014 [34]	**Risk of bias** Patient selection *(Unclear)*: unclear how the patient population was recruited. Index test *(Unclear)*: It was not specified if the index test was completed without knowledge of the reference standard results. **Applicability concerns** Patient selection *(high risk)*: the patient population consisted of those with “severe sciatica” and therefore not representative of those with mild and moderate symptoms. Reference standard (*Unclear*): unclear as there is no clear gold standard for diagnosing NP in LBLP
Vroomen et al., 2002 [19]	**Applicability concerns** Reference standard (*Unclear*): unclear as there is no clear gold standard for diagnosing NP in LBLP
Walsh et al., 2009 [14]	**Risk of bias** Reference standard *(high risk)*: The reference standard was a neurodynamic test which has been found to have low diagnostic validity. Flow and timing (*high risk*): the SLR and slump test were performed immediately after the nerve palpation which may have affected the test findings. **Applicability concerns** Reference standard (*Unclear*): unclear as there is no clear gold standard for diagnosing NP in LBLP

## Conclusion

Low-moderate level evidence supports the diagnostic utility of patient history, clinical examination and screening tool data to identify NP in LBLP. Issues relating to the quality of evidence are largely due to methodological flaws and issues regarding applicability of the included studies. The most promising diagnostic tools highlighted in this review include a cluster of eight patient history/clinical examination signs and the StEP tool.

Recommendations for low RoB and high level of evidence diagnostic utility studies have been made. Furthermore, a need for consistency in the use of terminology relating to NP in LBLP and a common reference standard to identify NP in LBLP is needed in order for stronger recommendations to be made.

Box 1: Example of MEDLINE OvidSP search strategy 1948 – July 2019 1. exp. “Sensitivity and Specificity”/ or Diagnostic accuracy.mp.

2. diagnostic utility.mp.

3. exp. “Reproducibility of Results”/ or exp. “Sensitivity and Specificity”/ or diagnostic reliability.mp.

4. 1 or 2 or 3

5. patient history.mp.

6. patient interview.mp.

7. subjective history.mp.

8. subjective examination.mp.

9. physical examination.mp. or exp. Physical Examination/

10. physical testing.mp.

11. objective examination.mp.

12. objective history.mp.

13. clinical examination.mp.

14. clinical testing.mp.

15. case ascertainment tool$.mp.

16. screening tool$.mp.

17. questionnaire$.mp. or exp. “Surveys and Questionnaires”/

18. 5 or 6,7,8,9,10,11,12,13,14,15,16,17

19. 4 and 18

20. neuropathic pain.mp. or exp. Neuralgia/

21. radicular.mp. or exp. Radiculopathy/ or exp. Intervertebral Disc Displacement/ or exp. Spinal Nerve Roots/

22. exp. Sciatic Neuropathy/ or exp. Sciatic Nerve/ or sciatic$.mp.

23. 20 or 21 or 22

24. 19 and 23

25. low back pain.mp. or exp. Back Pain/ or exp. Low Back Pain/

26. low back related leg pain.mp. or exp. Sciatica/

27. 25 or 26

28. 24 and 27

## Data Availability

No patient data sets used in this review. All data analysed in this review are included in the study.

## Abbreviations

NP: Neuropathic pain
LBLP: Low Back Related Leg Pain
NICE: National Institute for Health and Care Excellence
PRISMA-P: Preferred Reporting Items for Systematic Reviews and Meta-Analysis-Protocols
SPIDER: Sample, Phenomenon of Interest, Design, Evaluation, Research Type
LRs: Likelihood ratios
PPVs: Positive predictive values
QUADAS-2: The Quality Assessment of Diagnostic Accuracy Studies 2
RoB: Risk of Bias
GRADE: The Grading of Recommendations, Assessment, Development and Evaluations
SLR: Straight leg raise
SQST: Standardised qualitative sensory testing
MRI: Magnetic resonance imaging
S-DN4: Self-completed douleur neuropathique 4
PDQ: painDETECT questionnaire
S-LANSS: Self-completed Leeds Assessment of Neuropathic symptoms and Signs
CLBP: Chronic low back pain
CES: Cauda equina syndrome
HE: Hyper-extension
